# Protein conformational entropy is not slaved to water

**DOI:** 10.1038/s41598-020-74382-5

**Published:** 2020-10-16

**Authors:** Bryan S. Marques, Matthew A. Stetz, Christine Jorge, Kathleen G. Valentine, A. Joshua Wand, Nathaniel V. Nucci

**Affiliations:** 1 Johnson Research Foundation and Department of Biochemistry and Biophysics, University of Pennsylvania, Philadelphia, PA USA; 2 Department of Physics and Astronomy and Department of Molecular and Cellular Biosciences, Rowan University, 201 Mullica Hill Road, Glassboro, NJ 08028 USA; 3 Present Address: Department of Biochemistry and Biophysics, Texas A&M University, College Station, TX 77845-2128 USA

**Keywords:** Biophysics, Molecular biophysics, Thermodynamics, Solution-state NMR, Biophysical chemistry, Proteins, NMR spectroscopy

## Abstract

Conformational entropy can be an important element of the thermodynamics of protein functions such as the binding of ligands. The observed role for conformational entropy in modulating molecular recognition by proteins is in opposition to an often-invoked theory for the interaction of protein molecules with solvent water. The “solvent slaving” model predicts that protein motion is strongly coupled to various aspects of water such as bulk solvent viscosity and local hydration shell dynamics. Changes in conformational entropy are manifested in alterations of fast internal side chain motion that is detectable by NMR relaxation. We show here that the fast-internal side chain dynamics of several proteins are unaffected by changes to the hydration layer and bulk water. These observations indicate that the participation of conformational entropy in protein function is not dictated by the interaction of protein molecules and solvent water under the range of conditions normally encountered.

## Introduction

It is well established that water is a fundamental determinant of the structure and thermodynamic character of globular protein molecules, especially through the hydrophobic effect that arises from changes in the entropy of solvent water as the protein folds^1^. The participation of single waters as integral structural elements of protein molecules has long been appreciated^2,3^. The dynamical aspects of solvent water also potentially have great relevance to a number of biological functions of proteins such as catalysis and molecular recognition. Accordingly, the nature of the surface hydration of folded protein molecules continues to be the subject of intense investigation^4^. In this context, the “solvent slaving” model^5^ is commonly employed as a framework for describing the coupling of solvent water and internal protein motion^4^. Solvent slaving posits that the majority of internal protein motion is dictated by various general structural and dynamic features of water. However, this view is seemingly at odds with the realization that fast internal protein motion is a manifestation of large reservoirs of conformational entropy that can greatly influence the thermodynamics of protein functions such as the binding of ligands^6^ and the inherent stability of the folded state^7^. Here we show that the conformational entropy of soluble proteins is largely independent of the features of solvent invoked by the solvent slaving model.

The solvent slaving view proposes that internal motions of proteins are directly dependent on bulk solvent motions (Class I motions) or dependent on those of the layer of water nearest the protein surface, namely hydration water (Class II motions)^5^. Substantial evidence indicates that large-scale and relatively long-timescale (ms–µs) collective motions such as reorientations of domains or large sections of backbone are dependent on solvent viscosity and are also modulated by molecular crowding^8^. Here we are mostly interested in the influence of water on the distribution of fast internal side chain motion, which reflects the rotameric entropy of proteins that is intimately connected to the thermodynamics of protein function^6,9–11^.

Nuclear magnetic resonance (NMR) spin relaxation can be used to assess the mobility of individual bond vectors within the molecular frame^12,13^. Recent work indicates that changes in conformational entropy of proteins can be quantitatively obtained through a dynamical proxy based on NMR-relaxation in methyl-bearing side chains^6,14–16^. There are numerous reasons for the success of this approach as described in detail elsewhere^11^. Briefly, the vast majority of conformational entropy is found in states that interconvert on the subnanosecond timescale and reporter methyl groups are sufficiently coupled to the motion of surrounding side chains to provide a quantitative proxy for residual conformational entropy of the entire protein molecule^11^. This NMR-based “entropy meter” has been demonstrated by simulation^16^ and confirmed by experiment^6^. Using the “entropy meter,” it has been found that conformational entropy is a highly variable and often important component of the thermodynamics governing protein–ligand interactions^6^. Thus, significant effects of water on the distribution of states that are reflected in fast internal motion could greatly impact the fundamental thermodynamics underlying protein function.

Here we use these NMR relaxation methods to examine the influence of various solvent conditions on the fast motion of methyl-bearing and aromatic side chains and the backbones of the proteins ubiquitin, maltose-binding protein (MBP) and malate synthase G (MSG). High viscosity glycerol/water mixtures and encapsulation within the dynamically impeded water core of a reverse micelle are used to probe the influence of water solvent dynamics on the protein internal motion. The reverse micelle also mimics the most extreme level of confinement that a protein might experience within the cell. Importantly, reverse micelle encapsulation also facilitates site-resolved measurement of hydration water dynamics^17,18^, which enables a direct comparison of the dynamics of the protein to the mobility of the water near the protein surface site-by-site.

The effect of increasing bulk solvent viscosity on the motion of methyl-bearing side chains of ubiquitin was explored using 0, 30 and 50% (v/v) glycerol-water solutions corresponding to viscosities of 1.0, 3.0 and 8.4 cP, respectively^19^. The NMR relaxation in methyl groups is interpreted using the Lipari-Szabo model-free order parameter (O^2^) which ranges from 0, indicating complete isotropic flexibility, to 1, corresponding to complete rigidity within the molecular frame^20^.

Employing standard tetrahedral geometry for the methyl group allows the determined order parameter to be reduced to that corresponding to motion of the methyl symmetry axis (O^2^_axis_). It is important to emphasize that this measure of flexibility is sensitive only to motions faster than overall reorientation of the protein^20^, which ranges from 4 to 25 ns depending on context (see below and Supplementary Table 1).

**Figure 1:**
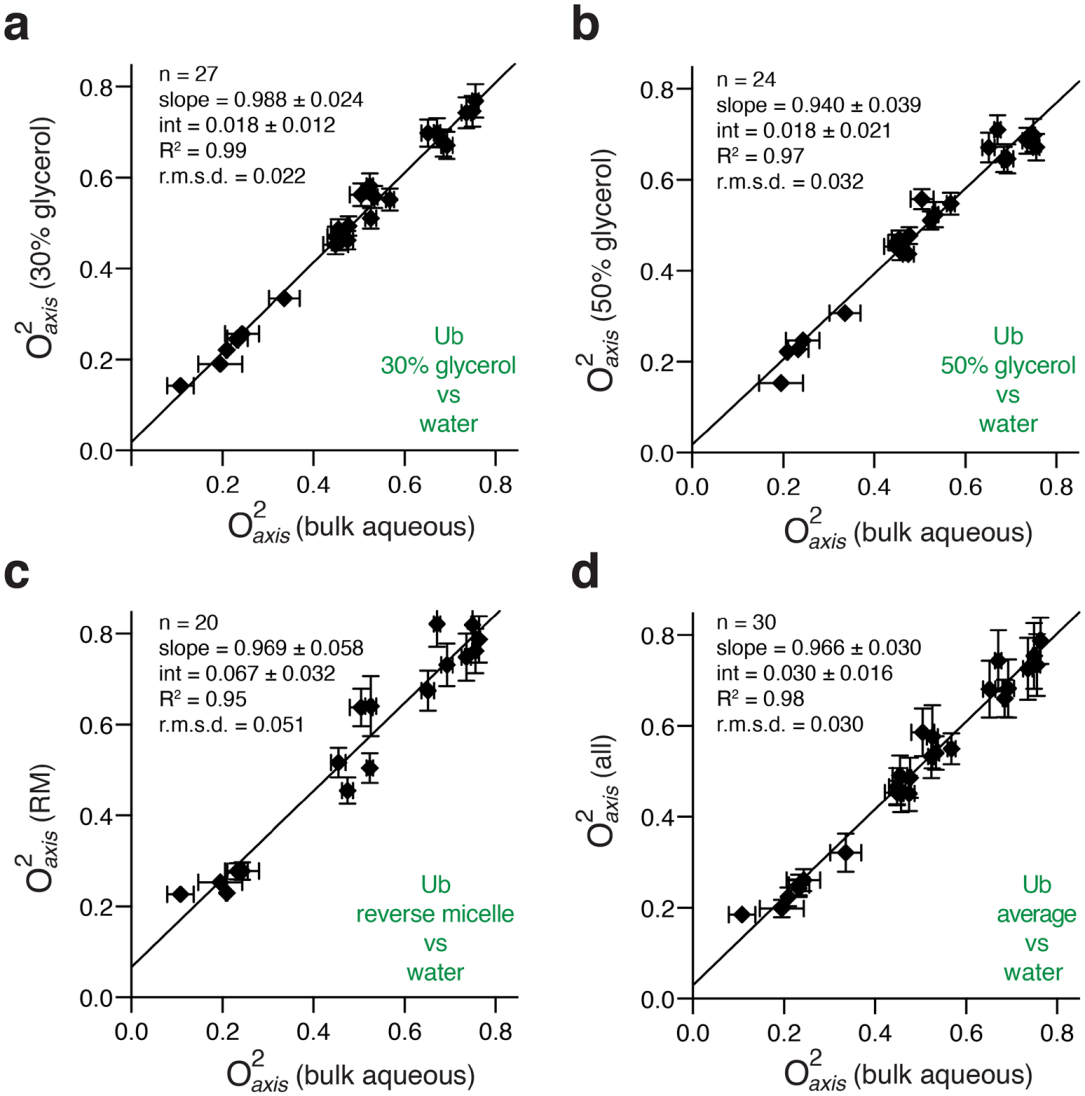
Influence of bulk solvent viscosity and confinement on fast methyl-bearing side chain motion in ubiquitin. Methyl symmetry axis order parameters (O^2^_axis_) of ubiquitin in high glycerol solutions and encapsulated in reverse micelles compared to those acquired in bulk aqueous solution^21^ (**a**) 30% (v/v) glycerol, (**b**) 50% (v/v) glycerol, (**c**) encapsulation in AOT reverse micelles, (**d**) average of methyl symmetry axis order parameters obtained in RM and the two glycerol solution conditions compared to bulk aqueous values. All data obtained at 25 °C.

We find that, in general, the motions of these probes are not influenced by the bulk solvent viscosity (Fig. 1). The average differences in O^2^_axis_ (< Δ O^2^_axis_ >) for ubiquitin in water and that in 30% glycerol and 50% glycerol solutions are − 0.013 ± 0.022 and 0.012 ± 0.020, which are within error of zero. Thus, there is no significant change in methyl-bearing side chain dynamics. Given the direct and quantitative connection between changes in methyl-bearing motion and changes in conformational entropy^11^, the absence of perturbation of fast methyl-bearing side chain motion when moving from one solvent condition to another means that there are no changes in the (corresponding) conformational entropy. In contrast, the solvent slaving model predicts that an increase in bulk viscosity should result in a general rigidification of these motions^5,22^, yet none is observed. It is important to note that the application^4^ of the solvent slaving model to proteins under physiological conditions is distinct from, though perhaps inspired by, the so-called low temperature “glass transition” that occurs around 200 K^23^. Even there, it should be noted that simple thermal cooling of side chain motion seen around room temperature predicts the low temperature transition^24^.

**Figure 2:**
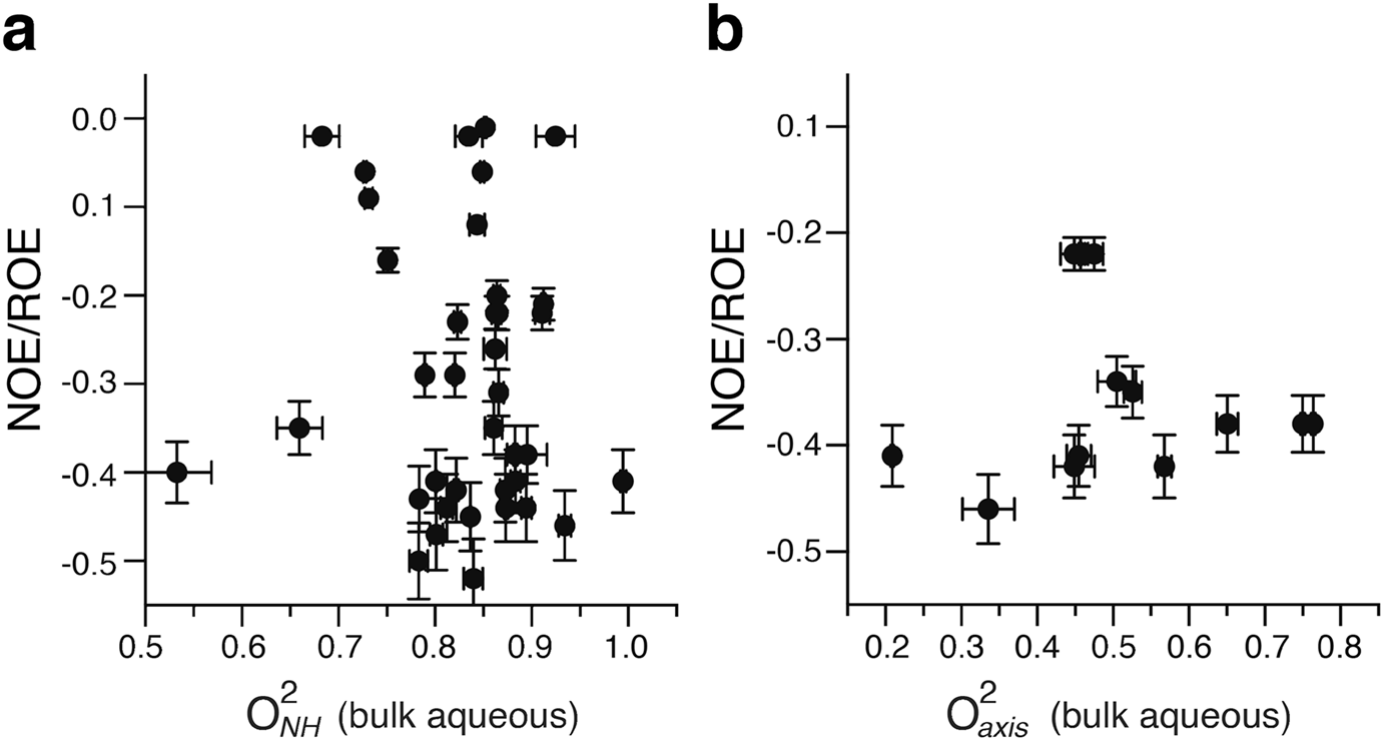
Fast backbone and methyl-bearing side chain motion in ubiquitin is uncorrelated with local hydration dynamics. Squared generalized order parameters of amide NH (O^2^_*NH*_) (**a**) and methyl symmetry axis (O^2^_axis_) (**b**) of ubiquitin are plotted against the local hydration dynamics at the nearest amide NH as measured by the ratio of the NOE and ROE between the amide and water for the protein encapsulated within a reverse micelle. See text for details. More negative values correspond to slower hydration water with a limit of −0.5 corresponding to water rigidly attached to the protein. Motion of water in the reverse micelle unassociated with protein core is fortuitously on the time scale of the null of the NOE (~ 100–300 ps). Only methyl groups within 5 Å of an amide NH are included in this analysis.

We then employed the unique properties of proteins encapsulated within the water core of reverse micelles to further investigate the influence of the dynamics of the hydration shell on internal protein motion. Under the water-limited conditions employed here, the reverse micelle encapsulates a single protein molecule surrounded by several thousand waters composing a 10–15 Å hydration layer^25^. The sign of nuclear Overhauser effects arising from protein-water ^1^H–^1^H dipolar interactions^26^ indicates that the effective correlation time of water adjacent to the protein in the reverse micelle is slowed by two orders of magnitude relative to bulk water^27^. Here, the reverse micelle serves two purposes: it eliminates the presence of bulk water while preserving the hydration layer (relevant to Class I) and greatly slows the motion of water in the first hydration layer (relevant to Class II)^28^. The motion of methyl-bearing side chains of ubiquitin confined to the water core of a reverse micelle are statistically indistinguishable from that of the protein in bulk solution (< Δ O^2^_axis_ >  = − 0.051 ± 0.036) (Fig. 1c). Furthermore, there is no apparent *general* correlation between local hydration dynamics at the surface of the protein and backbone or side chain motion (Fig. 2). These data collectively demonstrate that the conformational entropy of ubiquitin is largely independent of and certainly not dictated by either bulk or local hydration layer water dynamics.

**Figure 3:**
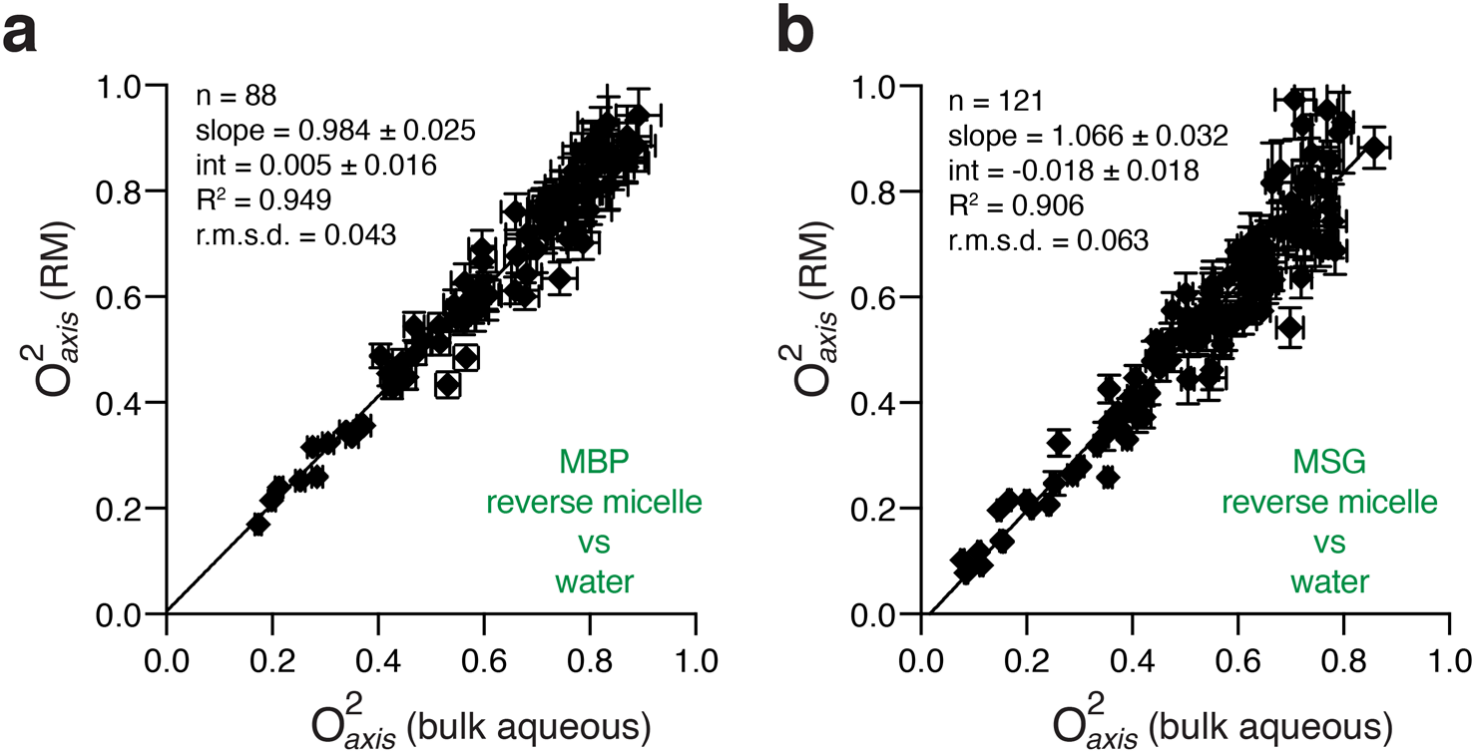
Methyl-side chain motion of proteins has little dependence on solvent dynamics or bulk solvent viscosity. Correlation plots of methyl order parameters (O^2^_axis_) for MBP (**a**) and MSG (**b**) in bulk aqueous solution and encapsulated within reverse micelles prepared with 150 mM deuterated cetyltrimethylammonium bromide (CTAB-*d*_*42*_) and 800 mM deuterated hexanol (*d*_*13*_) in perdeuterated pentane at molar ratios of water to CTAB (*W*_*0*_) of 15 and 22, respectively.

To test the generality of this conclusion we examined two larger proteins encapsulated in reverse micelles: maltose binding protein (MBP, 41 kDa) and malate synthase G (MSG, 81 kDa). Much like for ubiquitin, the methyl order parameters are essentially unaffected by encapsulation (Fig. 3). Furthermore, there is no correlation of the degree of disorder of a methyl group, as represented by its order parameter, with its depth of burial (Supplementary Fig. 3). Taken together it is clear that the Class I and II couplings of solvent with side chain motion demanded by the solvent slaving theory are absent at room (physiological) temperature. Correspondingly, in this context, the conformational entropy is independent of solvent.

**Figure 4:**
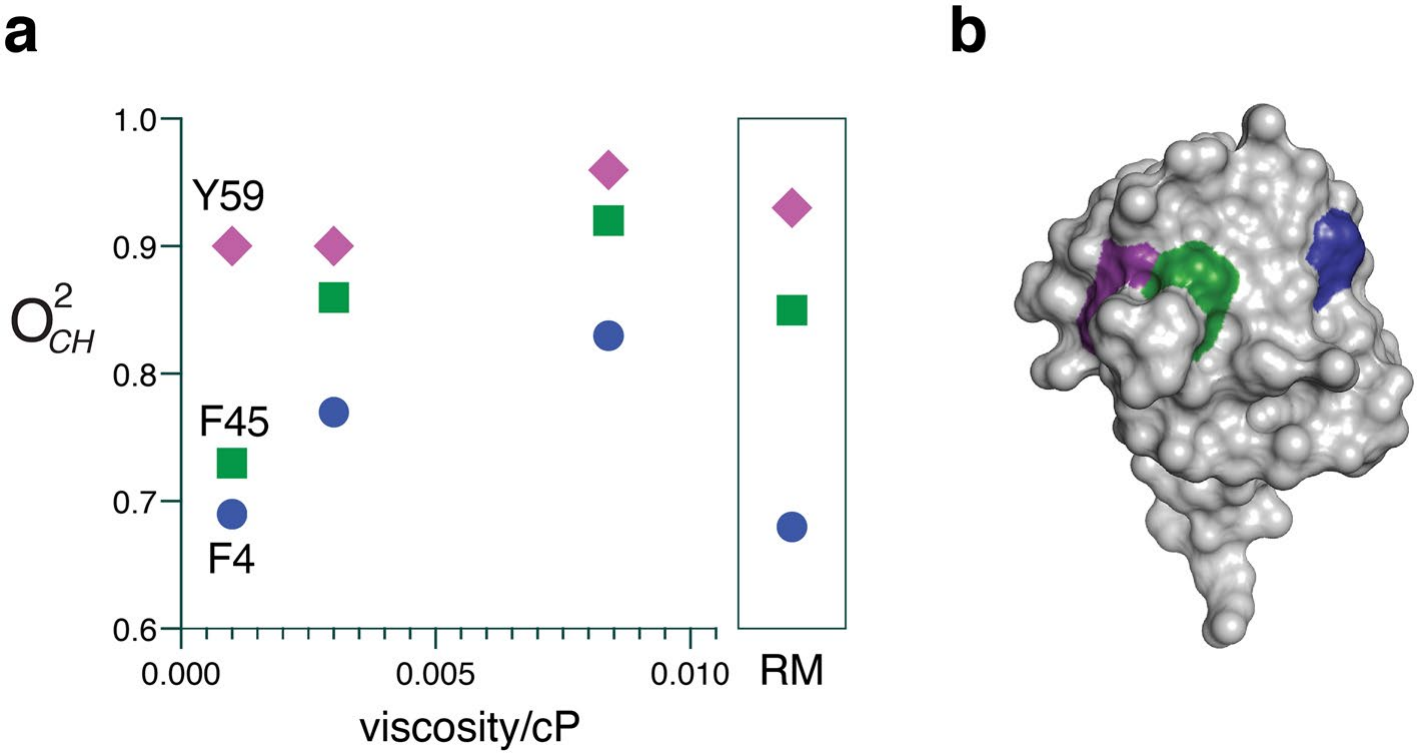
Influence of solvent viscosity and confinement on aromatic side chain mobility in ubiquitin. (**a**) The order parameters (O^2^_CH_) of F4 (blue circles), F45 (green squares), and Y59 (magenta diamonds) generally increase with increasing bulk solvent viscosity (bulk aqueous, 30% (v/v) glycerol, and 50% (v/v) glycerol, respectively). The dynamics of the aromatic rings of the protein encapsulated in a reverse micelle (boxed inset) are similar to that observed in bulk water consistent with the effect originating in the hydration layer. See text. (**b**) Rendering of the surface of ubiquitin with the contributions by F4 (blue), F45 (green) and Y59 (magenta) indicated. Surface drawn with PyMol (Schrödinger, LLC).

Some aspects of fast aromatic ring motion in ubiquitin are sensitive to the nature of the surrounding solvent. Ubiquitin contains three partially solvent-exposed aromatic side chains that have been shown to undergo limited ring rotation on the nanosecond time regime at the 293 K temperature used here^29^. Increasing the temperature above ~ 315 K brings full ring rotation into the subnanosecond time regime while application of hydrostatic pressure has little influence on ring dynamics indicating a low activation volume for motion on the ps-ns timescale^29^. Slow ms timescale ring “flipping” is often associated with high activation volume^30^ whereas faster motion^31^ with lower activation volume^29^ is suggestive of more continuous motion. These properties indicate that the interior of the protein is relatively liquid-like and that motion is diffusive and does not involve creation of significant unoccupied volume^29^. Here we find that the motion of the three aromatic rings of ubiquitin is rigidified on the nanosecond time scale by the presence of glycerol but to varying degrees (Fig. 4 and Supplementary Table 1).

Y59 is already quite rigid in bulk water and becomes only slightly more so in the presence of 50% (v/v) glycerol. F4 and F45 are significantly rigidified in the presence of 50% glycerol though to different degrees (ΔO^2^_CH_ = 0.14 ± 0.05 and 0.19 ± 0.09 for F4 and F45, respectively). Because glycerol undoubtedly penetrates the hydration layer, it is unclear whether these effects are related to bulk and/or hydration water. It has been shown that appropriate encapsulation of ubiquitin in a reverse micelle largely preserves the thickness of the hydration layer while stripping away bulk solvent^28^. Notwithstanding that the dynamics of the hydration layer are slowed in the reverse micelle, the motions of all three aromatic rings are roughly aligned with that observed in bulk water. This observation is consistent with that obtained by site-resolved tryptophan fluorescence relaxation^32,33^ and creates a view where ring rotation is coupled to a small diffusive structural excursion.

**Figure 5:**
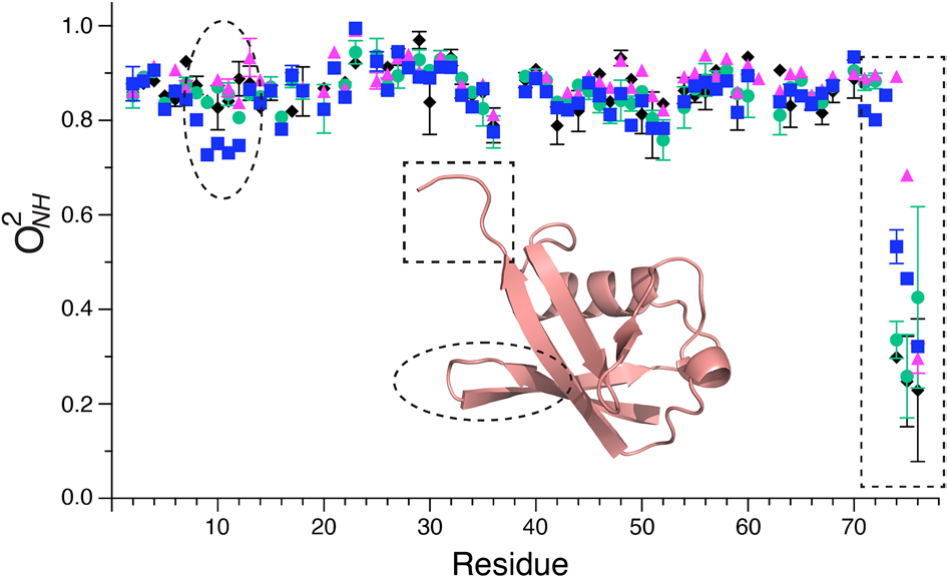
Influence of solvent viscosity and dynamics on the amplitude of fast motion of the backbone of ubiquitin. Dependence of amide N–H bond vector model-free squared generalized order parameter of the amide N–H (O^2^_NH_) at 20 °C on various solvation conditions are shown as a function of sequence position when the protein is in bulk aqueous solution (blue circles); encapsulated within the water core of AOT reverse micelles (magenta triangles); in bulk 30% glycerol (green circles) and 50% glycerol (black diamonds). Inset shows a ribbon representation of the ubiquitin with dashed lines indicating regions of slower ms-μs motion. See text. Ribbon representation drawn with PyMol (Schrödinger, LLC).

The mobility of the ubiquitin backbone was also evaluated under each solvent condition (Fig. 5 and Supplementary Fig. 1). In bulk water, the backbone units of secondary structure are generally rigid as is typical of a well-structured and stable protein^34^. Prominent dynamical regions are the fully solvated C-terminal tail comprised of residues 70–76, which show progressively smaller amide NH generalized order parameters (O^2^_NH_) consistent with superposition of local motions about the backbone torsion angles^34^, and a reverse turn involving residues 9–12, which undergoes a collective motion on the ns-µs timescale^35^ as well as faster localized motion (Supplementary Table 1). This distinction is reflected in the dependence on solution conditions where the motion of the reverse turn involves a large element of structure while the localized motions along the C-terminal tail of the protein do not. The C-terminal tail is not generally rigidified by glycerol or confinement of the protein within a reverse micelle. In contrast, the reverse turn shows reduced motion on the subnanosecond timescale. This behavior is a reflection of slowing of the motion into a time frame that the order parameter is insensitive to i.e. slower than overall tumbling of the protein (Supplementary Fig. 2). Because the rotational time of the protein is increased in the reverse micelle and glycerol samples, there is potential for an enhanced convolution of the local N–H motions of residues in the reverse turn with this collective motion. Solvent effects on collective motions have been reported in many systems^36^. Elsewhere in the protein the backbone motions are only slightly modulated by changes in solvent dynamics and viscosity (Supplementary Fig. 1).

In conclusion, the data presented here indicate that the conformational entropy of well-folded soluble proteins is largely independent of water at physiological temperature. This finding resolves an important but hitherto ill-defined attribute of the described hierarchies of protein motions^5,22,37^: protein conformational entropy is anticipated to be independent of intracellular locale and is thereby free to convey entropically-mediated functional responses^6,10,38,39^.

## Methods

Isotopically labeled ubiquitin was prepared^40^ using the following labeling schemes: uniformly ^15^N-labeled for backbone relaxation; uniformly ^12^C–^2^H, ^15^N–^1^H labeled for hydration measurements; and selective *ortho*-^13^C–^1^H aromatic labeling for aromatic carbon relaxation^27,41^. Aqueous and water/glycerol samples were prepared at 1 mM total protein concentration with 50 mM sodium acetate, pH 5.0, and 50 mM sodium chloride. Samples were prepared using mixtures of appropriately labeled ubiquitin such that backbone measurements could be carried out on each sample to determine the optimal tumbling model for calculation of order parameters. The ubiquitin reverse micelle sample was prepared^42^ in 75 mM bis-2-ethylhexylsulfosuccinate (AOT) at a *W*_0_ (molar water:surfactant ratio) of 10 in perdeuterated hexane. When encapsulation of each protein is appropriately optimized^25^, the chemical shifts of backbone and methyl resonances of the encapsulated protein are statistically indistinguishable from the protein in bulk solution indicating that the native state is maintained. Indeed, the explicitly determined structure of encapsulated ubiquitin is essentially that same as that in bulk solution and in the crystal^42^.

For all methyl dynamics experiments, isotopically labeled ubiquitin, β-cyclodextrin-bound MBP and MSG were prepared^28^ with selective^13^ CH_3_ methyl labeling^43^ for isoleucines (δ_1_ only), valines, leucines, and methionines (for MSG only) with uniform background deuterium and ^15^N labeling throughout. Bulk aqueous/glycerol samples were prepared at concentrations of 1 mM (Ub), 750 µM (MBP), and 800 µM (MSG). Reverse micelle samples for MBP and MSG were prepared with 150 mM deuterated cetyltrimethylammonium bromide (CTAB-*d*_*42*_) and 800 mM deuterated hexanol (*d*_*13*_) in perdeuterated pentane at *W*_0_ of 15 and 22, respectively.

NMR experiments were performed at 11.7, 14.1 or 17.6 T using Bruker Avance III spectrometers equipped with cryoprobes. Backbone and aromatic relaxation data were collected at 20 °C. Relaxation time constants were determined with nine relaxation times (three in duplicate for error estimation) with the exception of ^15^N T_2_ measurements in 50% glycerol for which six relaxation times (two in duplicate) were measured. Backbone ^15^N–H relaxation experiments were collected using either standard or TROSY-based^44^ (50% glycerol condition only) T_1_, T_2_, and heteronuclear ^1^H–^15^N NOE pulse sequences. Aromatic relaxation measurements were performed at two magnetic field strengths using ^13^C–H T_1_ and T_1ρ_ relaxation pulse sequences implemented as pseudo-2D experiments. Hydration dynamics measurements were collected as previously described^27^.

Intra-methyl proton-proton cross-correlated spin relaxation experiments were collected at 25 °C at 14.1 T (ubiquitin) or 17.6 T (MBP and MSG). For the reverse micelle and glycerol samples, gradient-selected echo anti-echo quadrature selection was used to minimize streaking from the background ~ 2% protonated solvents and (for ubiquitin only) from the protonated AOT surfactant molecules. Molecular tumbling times were determined^45^ using TROSY-detected T_1_ and T_1ρ_ experiments collected at two fields as described above. NMR data was processed with NMRPipe^46^ and visualized with NMRFAM-Sparky^47^.

Model-free calculations were performed using in-house software^48^. Optimal tumbling models (isotropic, axially symmetric, or anisotropic) and rotational correlation times were determined^49^ for each sample. Order parameters and τ_e_ values were determined using a grid search approach^50^, and errors were estimated using Monte Carlo. Details of these calculations are provided elsewhere^48^. Methyl O^2^_axis_ values were derived from measured order parameters under the assumption of tetrahedral geometry (O^2^/0.111). Backbone order parameters shown in the main text are the average of at least two replicate samples. Methyl and all aromatic order parameters are reported from a single sample. The precision of measured order parameters is generally better than a few percent^48^.

Data from ill-resolved methyl crosspeaks were removed for all analyses. Spectra of the ubiquitin and MSG reverse micelle samples had considerable streak artifacts from background protonated solvent (~ 2%) and from protonated surfactant molecules (ubiquitin only) (Supplementary Fig. 4). Crosspeaks within 40 Hz of the streaks (indicated by green dashed lines in Supplementary Fig. 4) were discarded (red circles in Supplementary Fig. 4). Spectra of encapsulated MBP did not suffer from these artifacts due to its high encapsulation efficiency and the availability of highly deuterated surfactant (Supplementary Fig. 4b).

The depth of burial was determined for given structural elements using Depth 2.0^51^ using the reverse micelle-encapsulated ubiquitin structural ensemble 1G6J^42^ (conformer 25 of 32), β-cyclodextrin bound MBP (1DMB^52^) and apo MSG (2JQX^53^). Structural images were created using PyMOL (The PyMOL Molecular Graphics System, Version 2.0 Schrödinger, LLC).

## Supplementary information

Supplementary information1
