# Supplementary material for: Protein conformational entropy is not slaved to water
Source: Sci Rep. 2020 Oct 16;10:17587. doi: 10.1038/s41598-020-74382-5 (PMC7567893; doi:10.1038/s41598-020-74382-5)
Supplement: Supplementary file 1 — Supplementary information1 [file 41598_2020_74382_MOESM1_ESM.pdf]

Supplementary Material

Protein conformational entropy is not slaved to water

Bryan Marques[1], Matthew A. Stetz[1], Christine Jorge[1], Kathleen G. Valentine[1],

A. Joshua Wand[1*‡] and Nathaniel V. Nucci[2*]

Author affiliations

[1]Johnson Research Foundation and Department of Biochemistry and Biophysics, University of Pennsylvania, Philadelphia, Pennsylvania, USA: Bryan S. Marques, Matthew A. Stetz, Christine Jorge, Kathleen G. Valentine, and A. Joshua Wand

[2]Department of Physics & Astronomy and Department of Molecular & Cellular Biosciences, Rowan University, 201 Mullica Hill Road, Glassboro, New Jersey, 08028 USA

*Corresponding author*s

Nathaniel V. Nucci (nucci@rowan.edu), A. Joshua Wand (wand@tamu.edu)

[‡] Present address: Department of Biochemistry & Biophysics, Texas A&M University, College Station, Texas, USA 77845-2128

Supplementary Table I. Fast aromatic side chain motion in ubiquitin under various solvation conditions.

	Temp	Viscosity	O^2_{CH}			τ_e (ps)		
Bulk Solvent	°C	η (Pa-s)	F4	F45	Y59	F4	F45	Y59
Water	20	0.0010	0.69 (0.05)	0.73 (0.085)	0.90 (0.031)	99 (5)	59 (20)	42 (27)
30% Glycerol	20	0.0030	0.77 (0.012)	0.86 (0.051)	0.9 (0.061)	73 (10)	220 (100)	334 (198)
50% Glycerol	20	0.0084	0.83 (0.021)	0.92 (0.034)	0.96 (0.007)	322 (68)	680 (355)	774 (42)
Pentane[2] (RM)	20	0.0002	0.68 (0.005)	0.85 (0.029)	0.93 (0.006)	100 (11)	157 (23)	86 (27)
Water[3]	50	0.0005	0.51 (0.002)	0.38 (0.015)	0.58 (0.014)	456 (4)	572 (2)	376 (4)
30% Glycerol	50	0.0014	0.87 (0.003)	0.92 (0.002)	1 (0.002)	973 (8)	980 (3)	1134 (33)

[1]Viscosities for water and pentane are as reported in the NIST webbook. For glycerol solutions, viscosities were calculated based on the volume ratio of glycerol to water[19].

[2]While pentane is the bulk solvent for the reverse micelle condition, it is important to note that the local viscosity *inside the reverse micelle* is comparable to the high viscosity glycerol solutions.

[3]Bulk aqueous data at 50 °C is from Kasinath et al[29].

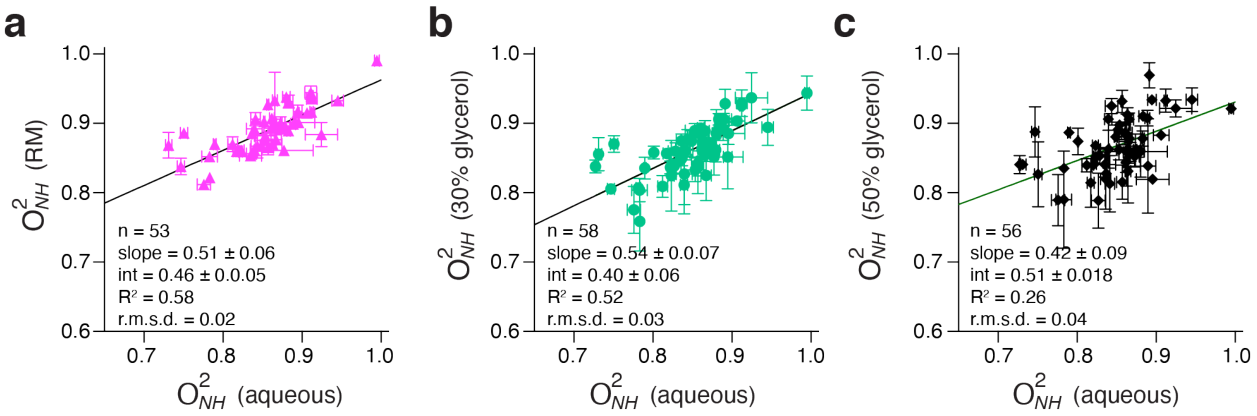

Supplementary Fig. 1 Correlation of O^2_{NH} obtained under RM (**a**), 30% v/v glycerol (**b**) and 50% glycerol solutions (**c**) with O^2_{NH} in bulk aqueous solution.

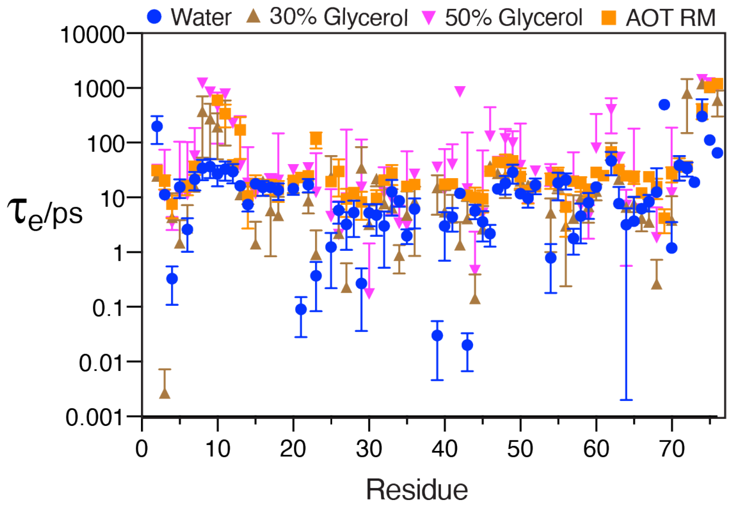

Supplementary Fig. 2 Influence of solvent viscosity and dynamics on the time scale of fast motion of the backbone of ubiquitin. Dependence of amide N-H bond vector model-free effective correlation times (τ_e) at 20 °C on various solvation conditions: in bulk aqueous solution (blue circles); encapsulated within the water core of AOT reverse micelles (orange squares); in bulk 30% glycerol (brown triangles) and 50% glycerol (magenta inverted triangles).

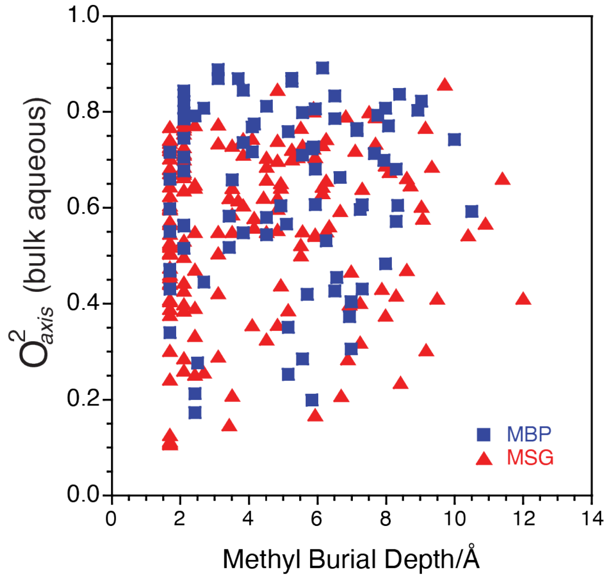

Supplementary Fig. 3 Lipari-Szabo methyl symmetry axis order parameters are not dependent on depth of burial. The methyl order parameters (O^2_{axis}) of aqueous MBP (blue squares) and MSG (red triangles) are plotted against the methyl burial depth as determined by the Depth 2.0 program[51]. There is clearly no correlation between methyl order parameter and probe burial depth, further demonstrating that protein conformational entropy is not slaved to solvent.

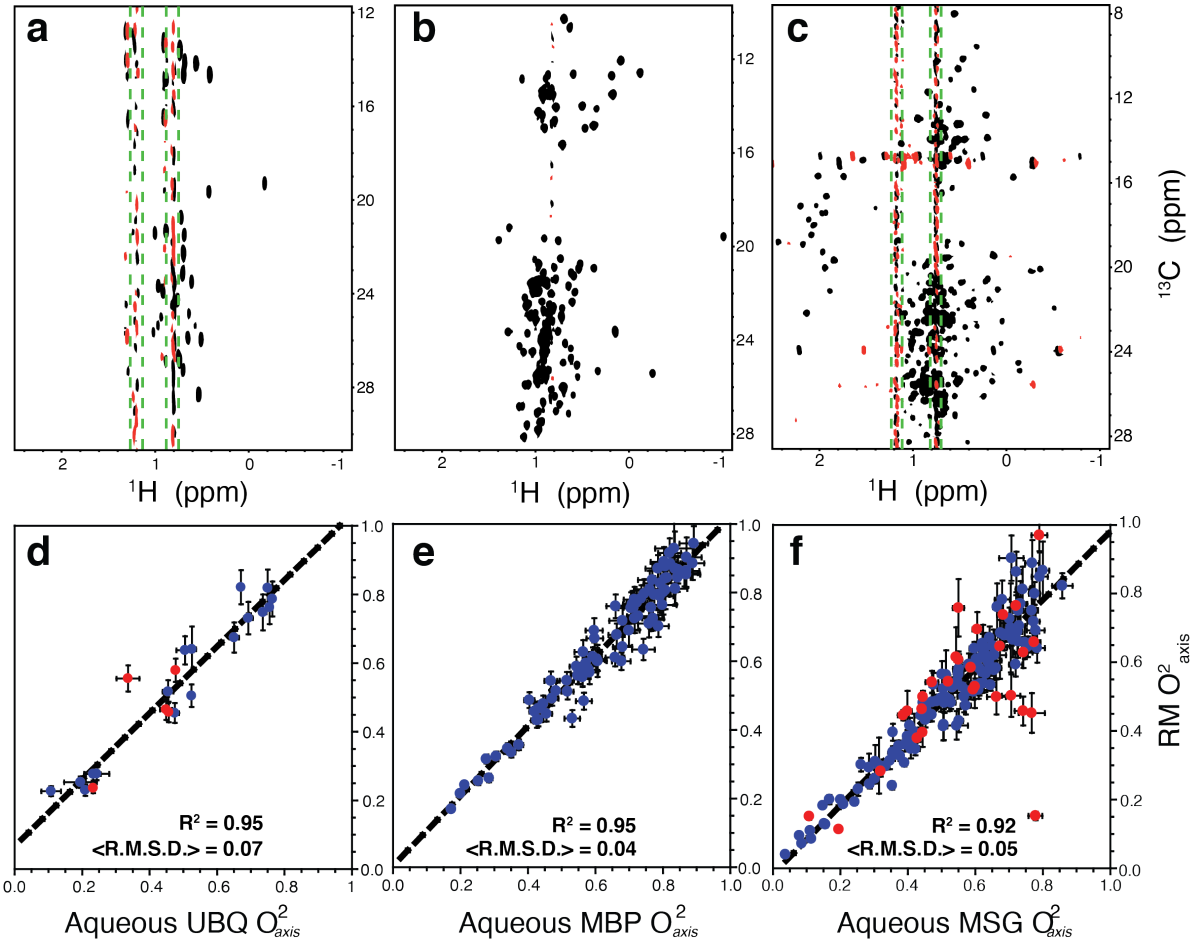

Supplementary Fig. 4 Streaking in the methyl region of ^{15}N- and ^{13}C-correlation spectra of proteins encapsulated in reverse micelles due to residual solvent protonation and protonated AOT surfactant molecules (ubiquitin only). ^{1}H-^{13}C HMQC correlation spectra derived from the shortest relaxation delay of the triple-quantum cross-correlated methyl dynamics experiment for ubiquitin **(a)**, MBP **(b)**, and MSG **(c)**. Streaking is observed at ~1.2 p.p.m. and ~0.8 p.p.m in the ^{1}H dimension for the ubiquitin and MSG spectra. Spectra of MBP are relatively uncompromised due to high encapsulation efficiency and the use of perdeuterated surfactants. Squared generalized order parameters of the methyl symmetry axis values of reverse micelle-encapsulated ubiquitin correlated with those obtained for ubiquitin (d), MBP (e) and MSG (f) in bulk aqueous solution. Red circles represent methyl probes within ~40 Hz of prominent streaks in the proton dimension (green dashed lines in **a** and **c**) and are excluded from the analyses.
